# The challenges experienced by Ukrainian refugees accessing General Practice: a descriptive cross-sectional study

**DOI:** 10.1093/fampra/cmaf012

**Published:** 2025-03-19

**Authors:** Niall O’Reilly, Emmet Smithwick, Eoin Murphy, Aisling A Jennings

**Affiliations:** Cork Postgraduate Specialist GP Training, St Mary’s Health Campus, 144 Templeacre Avenue, Cork, T23 Y7KN, Ireland; Cork Postgraduate Specialist GP Training, St Mary’s Health Campus, 144 Templeacre Avenue, Cork, T23 Y7KN, Ireland; Cork Postgraduate Specialist GP Training, St Mary’s Health Campus, 144 Templeacre Avenue, Cork, T23 Y7KN, Ireland; Department of General Practice, University College Cork, Room 2.41 Western Gateway Buiding, Cork, T12 XF62, Ireland

**Keywords:** refugees, asylum seeker, Ukrainian, General Practice, healthcare access

## Abstract

**Background:**

The war in Ukraine has led to an influx of Ukrainian refugees across Europe. Internationally, there is limited research into refugees’ experiences of accessing Primary Care. Furthermore, few studies have explored the experience of one homogenous refugee group. No study has explored the specific experience of Ukrainian refugees. To improve the care provided to this marginalized group it is important to understand the challenges they experience. The aim of this research is to identify the barriers Ukrainian refugees experience when accessing General Practice in Ireland.

**Methods:**

A 63-item questionnaire was distributed via Ukraine Action Ireland, a registered charitable organization, to Ukrainian refugees in Ireland. Qualitive comments were collected through free-text responses and were analysed using thematic analysis.

**Results:**

A total of 368 questionnaires were completed. About 75.4% of respondents reported that they were not asked about their mental health during consultations with their GP. About 25% of respondents could not attend GP due to transport difficulties. About 55% of respondents reported that a translator was needed but only one-third of respondents reported that one was offered. Self-reported health was relatively poor when compared with refugees in other countries and with Irish citizens. Three themes were developed; disparity in patient autonomy, perceived disregard for the refugee experience, and challenges in health care access.

**Conclusion:**

At a time of significant capacity challenges in General Practice it is paramount that resources are provided at a national level to address the challenges Ukrainian refuges currently experience.

**Key messages**

- First survey of Ukrainian refugees’ experiences of general practice.
- Ukrainian Refugees are older, predominantly female compared to other refugee groups.
- About 75.4% of respondents were not asked about their mental health during GP consultation.
- About 76% of respondents did not know about the out-of-hours GP services.
- Healthcare systems differences between Ukraine and the host country—barrier to access.

## Introduction

The war in Ukraine which began in 2014 and escalated to a full-scale invasion in 2022, has led to an influx of Ukrainian refugees to Ireland. According to Central Statistics Office in Ireland, there were 104 870 Personal Public Service Numbers given to arrivals from Ukraine between March 2022 and February 2024, under the Temporary Protection Directive [1]. Like many countries, general practice in Ireland faces significant capacity challenges at present [2]. Previous research conducted in the UK has shown that General Practitioners (GPs) who are already at capacity, find the needs and expectations of refugees difficult to fulfil [3]. This further contributes to the difficulty refugees experience accessing medical care [4]. Refugees are a vulnerable group that present with complex mental, physical, and social needs as a result of displacement, interrupted medical care, poor living conditions, and the traumatic experience of war [5]. Early and appropriate health care is essential. Unmet healthcare needs at refugees’ destination countries include language barriers, cultural misunderstanding, waiting times, financial constraints, transport difficulties, and a sense of stigma that have led to reduced symptom reporting and poor health outcomes [6, 7].

The differences in health care systems between the country of origin of the refugee group and the host country can be problematic. The Ukrainian healthcare system is based on a Soviet-era model which gives patients direct access to secondary care even with minor conditions. A network of GPs was emerging prior to the war but this was still in its infancy [8]. The Ukrainian system is theoretically free but several reports show fees for doctors’ appointments, medicines, and investigations [9]. In Ireland, the Health Service Executive (HSE) is responsible for the delivery of health care. Approximately 30% of patients in Ireland, including all Ukrainian patients under the temporary care directive, have a medical card entitling them to medical care that is free at the point of care. This includes access to GPs, all hospital care, and medication [10, 11]. Eligibility for free GP care in Ireland is based on means testing and successful applicants are provided with a medical card. Unlike the Ukrainian model, in Ireland GPs have a gatekeeping role. Patients first point of contact with a health-related symptom is typically with their GP who will treat the patient or refer for hospital care, if necessary. Although hospital care is free in Ireland, waiting times in the public service can be long depending on the nature of the referral. Waiting times for private care are shorter but if a patient does not have private health insurance, the cost of these services can be prohibitive [11].

The Ukrainian population have distinct medical comorbidities, some of which are not routinely screened for in Ireland. These include one of the highest burdens of multi drug resistant Tuberculosis in the world, low rates of coronavirus (COVID) vaccination (approximately 35%), an ongoing  human immunodeficiency viruses (HIV) epidemic, and relatively low vaccination against measles and rubella [12]. There is a high prevalence of thyroid disease and malignancy as a result of the Chernobyl disaster of 1986. The use of alternative and herbal medicines is widespread [13]. The prolonged conflict in Eastern Ukraine has led in an increase in post-traumatic stress disorder (PTSD) [14]. Damage to medical facilities and disruption to supply chains has made access to essential medications difficult which further complicates the journey of refugees living with chronic disease [15]. Due to conscription, the journey from the Ukraine has been mainly made by women and children. The conditions experienced on the journey have impacted healthcare and exploitation has been reported [16].

There has been limited research into refugees’ experiences of primary healthcare and, no published research into the Ukrainian experience of General Practice in Ireland. Internationally, several studies have investigated the refugee experience of General Practice (5, 17, 18) but few have investigated the experience of one particular refugee group. This has been noted as a limitation in previous research as cultural issues are difficult to assess across a heterogenous refugee group [19]. To effectively provide care to this vulnerable group we need to understand the challenges Ukrainian refugees experience accessing general practice in Ireland thereby allowing us to direct policy and funding nationally as well as creating targeted interventions in general practice.

The aim of this research is to identify the barriers Ukrainian refugees experience when accessing General Practice.

## Methods

The study was approved by the ethics committee for the Irish College of General Practice (ICGP_REC_2022_T08).

### Study participants

Participants were Ukrainian refugees over the age of 18 years who arrived in Ireland after the temporary protection status began on the 24 February 2022. Participants were recruited by Ukraine Action Ireland (UAI), a registered charitable organization, through their social media platforms. The questionnaire was delivered using Kobotoolbox [20], an online software suite used to collect and manage data. Only one response was allowed per URL thus reducing the risk of repeat completion of the form by one individual. The questionnaire was available in both Ukrainian and English and the translation was performed by UAI. Translation of the questionnaire and responses was carried out by two separate translators.

The questionnaire included 63 questions across several subsections including health literacy, demographics, health status, access to the medical card, knowledge of healthcare systems, access to GP, and health beliefs. The items in the questionnaire were a mixture of previously validated [21] and unvalidated questions. The unvalidated items were essential to address the aim of the study. A structured approach was used when designing and grouping unvalidated questions [22].

Health literacy was established using a single-item screening tool ranked on a Likert scale [23]. Demographic information that was gathered included the county in Ireland that the participant currently lived in, marital status, age range, gender, and the nature of the participants residence in Ireland. Health status was assessed using a validated single item scale for self-rated health which was graded from very poor to very good [24]. Access to Healthcare, Unmet Health Needs and Knowledge of Healthcare systems were assessed based on a modified version of a previous study [25]. Participants’ experience of access to general practice was assessed using 18 questions designed *de novo*.

Three open-text questions at the end of the form allowed the participants the opportunity to describe their positive and negative experiences of healthcare in Ireland.

Data collection began on 26 February 2023 and was completed on 11 April 2023. The open text section of the collected questionnaires was then translated into English by translators provided by UAI.

### Data analysis

All data was collected and analysed using MS Excel. Three free-text boxes were included in the questionnaire. The response to these free-text comments were entered into an MS Word document, one document for each free-text comment box. NVivo 12 was used to manage the data. Free-text comments were analysed using thematic analysis employing an inductive approach as outline by Braun and Clarke [26]. The responses to each free-text comment were initially analysed separately before being considered as a whole. One researcher (NO’R) led the data analysis process with support from a second researcher (AJ), an experienced qualitative researcher. The two researchers compared, contrasted, and consolidated codes through a process of familiarization, identification, and discussion of the content of the free-text comment boxes. Codes that shared similar meaning were consolidated into sub theme which were subsequently developed, through a process of discussion with the wider research team, into overarching themes.

## Results

In total, 368 questionnaires were completed. 367 of the 368 respondents were Ukrainian nationals living in Ukraine who were displaced on or after 24 February 2022 and non-Ukrainian citizens who were living in Ukraine before 24 February with their Ukrainian family. One respondent was a refugee living in Ukraine prior to 24 February who was displaced to Ireland. 84% of the respondents were female and 71% were within the age range of 31 to 50 years. About 39.7% were divorced/widowed or single while 60.3% were married or in a civil partnership. 60.8% stated that they were with their partner in Ireland.

The majority of respondents reported their self-rated health as ‘good’ or ‘neither good nor poor’ on a Likert scale (Table 1). With regards to respondents’ understanding of the healthcare system (Table 2), the vast majority of respondents (91%) had received a medical card and the majority (81%) found the process of applying for a medical card to be ‘easy’. About 13% had been admitted to a hospital in the previous 6 months while 64.9% visited their GP.

**Table 1.** Self-reported Health.

How would you consider your health at the moment?	
Very good	13 (3.5)
Good	121 (32.9)
Neither	166 (45.1)
Poor	62 (16.9)
Very poor	6 (1.6)

**Table 2.** Understanding of Irish Health System.

Who explained the process of applying for a medical card to you?	*N* = 279
Friend/Relative	42 (15%)
Government authority	55 (19.7%)
Found on my own	114 (40.9%)
Medical Professional	22 (7.9%)
Other	46 (16.5%)
Why was the process of applying for the medical card^*^ difficult?	*N* = 61
It was not explained well	21 (34.4%)
Language difficulty	7 (11.5%)
Other	33 (54.1%)

^*^A medical card provides access to GP visits that are free at the point of care and also allow patients to access their medications for free. All Ukrainian refugees are entitled to this card.

The majority of patients located a GP based on HSE recommendations or other sources (Table 3). 76.1% of respondents were not refused access by a GP. Just over half (55%) of respondents reported that a translator was needed but only one-third of respondents reported that a translator was offered. In 35% of cases, a family member or child was reported to have been used as a translator. When asked about their relationship with their GP just over half of respondents (55%) reported they trusted their GP. Similar numbers of respondents (54%) reported that they trusted the treatment the GP initiated. When asked about consultations they had with their GP the majority of respondents (66%) felt that their GP listened to them. However, 37% of respondents felt like a burden on the service. Just under 10% of respondents reported they experienced discrimination from their GP. The majority of respondents (75%) reported that their GP did not ask about their mental health. When asked about the clinical examination component of the consultation, 56% of respondents felt that they were not examined properly and 88% felt that would have been examined differently in the Ukraine. With regards to secondary care referrals the majority of respondents (70%) were frustrated with the time it took to be reviewed in the hospital after their GP had referred them. Of note, 67% of respondents did not bring medical records from Ukraine.

**Table 3.** Access to GP.

	*N* (%)
How did you locate a GP? (*n* = 264)	
Google	24 (9.1)
Recommended by friend/relative	66 (25)
Recommended by HSE	81 (30.7)
Other	93 (35.2)
How many GPs did you contact before you were successful in getting a GP? (*n* = 264)	
1	189 (71.6)
2	24 (9.1)
3	22 (8.3)
4	8 (3)
5+	21 (8)
How many days after receiving a medical card were you accepted to a general practice? (*n* = 264)	
1	132 (50)
2	8 (3)
3	20 (7.6)
4	5 (1.9)
5+	99 (37.5)
Was it difficult to make an appointment with your GP (*n* = 328)	
No	184 (56.1)
If so, why was it difficult? (*n* = 144)	
Language barrier	18 (12.5)
No appointment availability	104 (72.2)
Other	13 (9)
Perceived discrimination	9 (6.3)
Do you feel that in your current living place, you or your family members have access to medical care when you are concerned for your health?	*N* = 368
Completely	90 (24.5%)
Moderately	167 (45.4%)
A little	59 (16%)
No	52 (14.1%)
How do you get to your GP?	*N* = 368
Friend/relative	17 (4.6%)
Personal car	62 (16.8%)
Public transport/walk	256 (69.6%)
Taxi	33 (9%)

**Figure 1.**
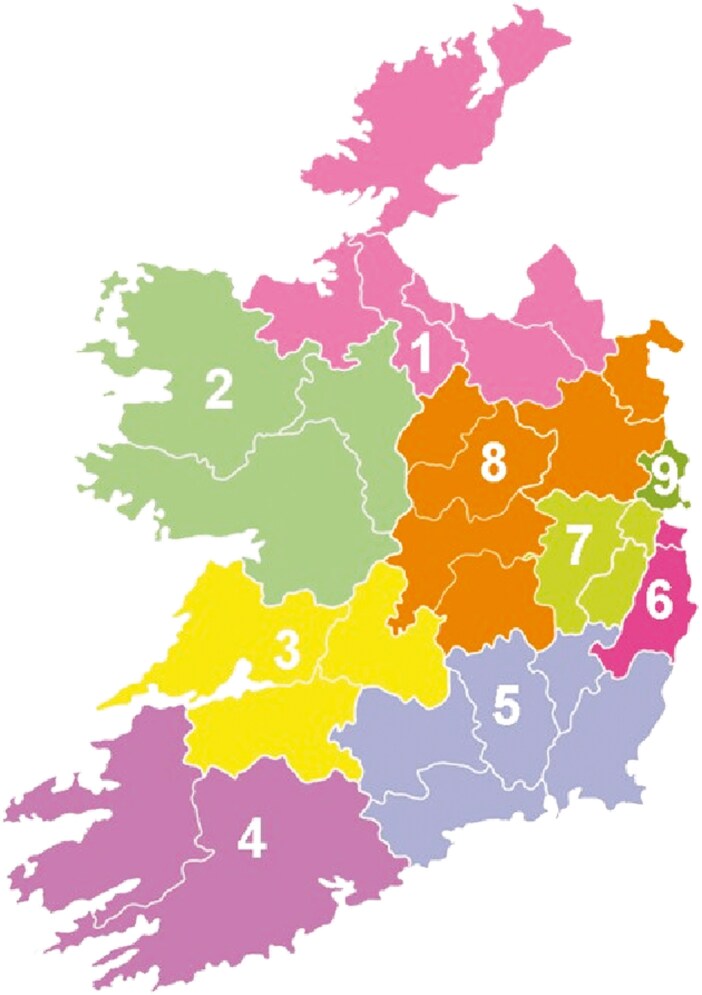
Knowledge of out of hours services by CHO.

To the question ‘Do you know about the out-of-hours GP/family Doctor service’, there were significant differences noted for community health organizations (CHO) 6 and 8 with only 8.3% and 4.9% of respondents, respectively, with knowledge of the out-of-hours services. Knowledge of out of hours in CHO 4 and 5 was higher than the national average (Fig. 1). 76% of respondents did not know about the out-of-hours GP services, 44% did not know how to access an ambulance and 58% did not know how to access an emergency department.

## Thematic analysis

The three main themes identified through thematic analysis include disparity in patient autonomy, perceived disregard for refugee experience, and challenges in access to healthcare.

### Theme 1—disparity in patient autonomy

A significant number of respondents experienced difficulty accessing their medical notes and results from their GP in Ireland. The fact that an appointment with a hospital specialist required a referral from a GP represented a new challenge for many patients from Ukraine. Many patients were fearful about their health as a result and described Ireland as having quite a paternalistic healthcare system.

*‘It would be good if the results of examinations and notes after a doctor’s appointment were available for viewing and saving by the patient, … the medical history must be with you’.*

*‘It is not convenient for me that everything is decided for me by the GP and I cannot influence the steps of my treatment without his approval’.*

### Theme 2—perceived disregard for the refugee experience

Respondents described experiences of an apathetic attitude from their GP and a perceived lack of patience from their GP due to language difficulties. Respondents also reported a perceived poor approach to treatment. These factors in conjunction with difficulty accessing GP care gave rise to suspicions of inequity in the system. Furthermore, for people with multiple comorbidities long periods between leaving Ukraine and a first appointment with a GP in Ireland lead to fear of disease progression and exclusion.

*‘I came to the doctor with a problem, he did not pay attention to my symptoms and said that I need to lose weight. When I told her about depression, he pretended that he did not understand me’.*

*‘Maybe it’s because our experience with Ukrainian doctors is different, Irish doctors often look unconcerned about patients. Because of lack of time, you always feel like they are rushing you’.*

However, many respondents felt that their care in general practice in Ireland was marked by kindness and politeness. A lack of corruption in the Irish healthcare system was noted several times.

### Theme 3—challenges in healthcare access

The third theme, which was the most dominant theme, was access to healthcare. This included access to medical notes, medicines, specialist services, GPs, transport, investigations, results, translators, and specifically access to Ukrainian Doctors. Waiting times were also discussed by many respondents.

*I do not have a GP due to the inability of doctors to accept new patients.*

*I speak English, but our doctor has a very strong accent, and a lot of medical terms are incomprehensible. But at the same time, I understand very well that the burden on doctors and the medical system of Ireland is very high now, and therefore it is difficult to meet all the needs of Ukrainians and not only them.*

*The remoteness of the ultrasound and X-ray rooms, it takes a long time to wait for the results of laboratory tests, in Ukraine we received the result after a few hours, here at least a week. Very long queues for specialists in a narrow field, such as gynaecologists, ENT.*

## Discussion

This study assessed the barriers experienced by Ukrainian refugees when accessing healthcare in Ireland. The results show that Ukrainian refugees, a predominantly female population group in Ireland, have relatively lower self-rated health when compared to other distinct groups of refugees or the Irish population. Although the majority of respondents received a medical card allowing them to access free healthcare 24.2% did not understand the function of the card. Furthermore, the language barrier created challenges for refugees when accessing appointments, reduced trust in the GP during the consult, and negatively affected the understanding of the health care system. The differences between the Irish and the Ukrainian health care systems were a source of frustration and distress for respondents, specifically, waiting times, a lack of access to medical notes, and no direct access to secondary care felt paternalistic to most respondents. Respondents reported that translators were not routinely offered, and mental health was not regularly assessed. Finally, proximity to the practice from their accommodation centres was a barrier to attendance.

## Strengths and limitations

This is the first research to survey Ukrainian refugees’ experiences of the health care system in Ireland and the first research into the Ukrainian refugee experience of primary care internationally. Previous research has highlighted the lack of research into the refugee experience of healthcare in their host country and specifically emphasized the need to explore the experience of one ethnic refugee group [27, 28]. This study addresses this gap in the literature.

The narrative available through the thematic analysis of the open-text responses adds insight and context to the quantitative element of the research.

The involvement of UAI was been a strength in that respondents trusted the source of the questionnaire. However, as a result all participants were recruited via social media channels which excluded people who were not connected with UAI thus affecting the sample frame. As the survey was exclusively online, a certain degree of computer literacy and may, therefore, have excluded some members of the Ukrainian refugee community.

Although there were 368 responses to our survey, it is difficult to establish the response rate of this study given the nature of the distribution. The study is population based, but the responding sample is self-selecting and the health status of the respondents is self-reported. As the study is cross sectional in design, the participants are subject to recall bias.

## Comparison with existing literature

The participants in this study are representative of the Ukrainian population in Ireland [1]. The respondents to this study were predominantly female which is likely due to conscription in the Ukraine. Research to date has found that female refugees are less likely to seek out healthcare which increases the risk of transition to poor health [29].

Respondents in this study had relatively lower self-rated health compared to the general population in Ireland [30]. The difference may be attributed to the mental stress of transit, poor access to healthcare and medicine on the journey from the Ukraine to Ireland and cultural and financial barriers on arrival [31]. However, a Canadian study has shown self-report health amongst the refugee population as higher than the general population in their first year of resettlement. This may be due to supports offered to refugees in the first year in Canada under the Resettlement Assistance Program [5] or the ‘Healthy immigrant effect’. This theory suggests that immigrants tend to be healthier than the general population in their host country upon arrival. Factors underpinning this phenomenon include the inability of the elderly or infirm to undertake arduous travel and the selection or younger immigrants for work permits [32]. The respondents to this study also reported lower self-rated health compared to a 2022 study of Syrian refugees in Ireland [21]. The cohort in our study was relatively older and predominantly female (84%) compared to the younger male cohort in the aforementioned study.

Research with refugees from several nationalities in both Scotland [27] and England [4]found that difficulty in understanding the host nations medical care system affected confidence in the system generally, in the GP consult specifically [18]. In our study a large proportion of respondents experienced difficulties finding a general practice that would accept them. The majority felt that this was due to a lack of availability. In the open text section, several respondents noted that their local general practice was no longer accepting patients as their lists were full. This is not a challenge that is unique to the refugee population in Ireland. The general population in Ireland are currently experiencing similar challenges accessing primary care due to the current GP shortages [33].

Respondents were frustrated by the challenges they experienced accessing both GP and secondary care in Ireland. The gatekeeper function of the GP in Ireland and the lack of patient access to shared medical records was perceived by respondents as a paternalistic approach to healthcare. Frustration with a lack of access to notes, investigations, and a lack of direct access to secondary care has also been reported in a previous study with Syrian refugees in Ireland [5, 21]. However, in this study, respondents appreciated access to free healthcare as, according to respondents, this was not routinely available in the Ukraine. Furthermore, in the free-text comments, several respondents spoke positively about the lack of ‘corruption’ in healthcare in Ireland. Long waiting times were specifically cited as a source of anxiety and led to a fear of disease progression. This finding is common for refugees coming from similar models of health care [4, 17]. The majority of participants reported that they trusted their GP and the treatment that the GP offered. Most participants felt that their GP listened to them. On the other hand, the majority of respondents in our study felt that they were not examined properly by their GP and felt that they would have been examined differently in the Ukraine. A lack of physical examination has been shown to create patient mistrust in their GP [17] and previous research with refugees has shown that a lack of examination can be perceived as discrimination by the patient [27]. Previous studies have also shown that refugees expected their doctor to take control of the consult and to tell the patient what was wrong. The open text response in this study yielded conflicting responses. While some respondents appreciated that doctors in Ireland were kind and polite others would have preferred a more direct approach [17]. A significant number of respondents felt that Ukrainian doctors living in Ireland as refugees should be allowed to treat the refugee population. Previous research has found that refugees tend to seek out native speakers as their physicians [34].

The majority of respondents to this study (75%) reported that they were not asked about their mental health. Depression and PTSD, related to violence, human rights violation, resettlement, and traumatic migration are well documented in the refugee population [35], as is the relative lack of screening for mental health disorders for refugees in their host countries [36–38]. Research to date reports that female refugees under report depression as they often feel rushed during a medical consult, do not have access to translators or simply were not asked about their mental health by their doctor [39, 40]. The majority of respondents to our study reported that a translator was needed, but only one third reported that a translator was offered. In the open text section, language difficulties were cited as a barrier to making an appointment and regarded as a source of perceived discrimination on the basis of refugee status. An inability to use translation services was perceived as apathy on the part of the GP. Several studies have shown that translation services are often unavailable when needed or inadequate for the needs of refugee populations [18]. Previous research has shown that the lack of an interpreter reduces the likelihood of further health education and health screening beyond the basic requirements of the consult [41]. Several respondents noted in the open text section that primary prevention and screening were not a feature of their consult.

Only one quarter of our respondents felt that they could access the medical care that they needed from their place of residence with the majority (70%) using public transport. A similar number of respondents were less likely to seek medical care due to distance from medical facilities. This may be due to the disproportionate placement of refugees in accommodation in rural parts of Ireland which may not have easy transport links to medical centres. Transport difficulties and proximity to healthcare services are an established barriers to access [42].

## Implications for policy, clinical practice, and research

GPs in Ireland are oversubscribed, and burnout is at high levels [43]. New training places are being added to postgraduate GP training schemes throughout the country to replenish the ageing GP workforce but it will take several years before the benefit of these increased numbers are realized [44]. The influx of Ukrainian refugees has placed a significant extra workload on rural practices in particular. To meet the increased workload created for these GP practices government funding should be made available for extra staff in medical, administration and nursing roles.

According to the Irish Medical Council, 280 Ukrainian doctors have signed up to NDTP HSE (National Doctors Training and Planning) which grants access to relevant courses and 50 have applied for professional registration, of which one doctor has been successful [45]. This process should be expedited and roles in advocacy and health promotion should be developed and made available for Ukrainian doctors and health care professionals living in Ireland. Trusted advocacy groups such as UAI should be included in health care promotion campaigns that target Ukrainian refugees and funding should be allocated accordingly.

An accessible digital resource that would simply and clearly explain the Irish healthcare system and provide regular updates should be developed. This would help to inform Ukrainian refugees about the structure of the Irish health care system, their entitlements to free care, and the resources that are available locally and nationally.

The appropriate use of translation services, thorough mental health assessment, and primary prevention should be promoted. Previous research has shown that only 33% of GPs in Ireland know how to access translation services and the majority felt that poor translation within the consultation had led to adverse outcomes for patients. Barriers to using translation services include waiting times for appointments with translators and a lack of expertise in specific dialects [46]. Quick reference guides and awareness campaigns targeted at GPs through medical periodicals and the governing body would help to raise awareness, improve health outcomes, and ultimately reduce the cost to the health service.

There are several opportunities for further research. An exploration of the difficulties that GPs experience providing care for Ukrainian refugees would complement the findings of this study. The perspective of the GP regarding specific questions raised by this research including the use of translation services, addressing patients’ expectations of the health service and in particular, mental health screening, and the use of referral pathways would help to further clarify the difficulties experienced by respondents to this study.

It is unclear when the conflict in Ukraine will be resolved. Longitudinal research into the physical and mental health of Ukrainian refugees is essential to tailor policy and funding in the interim.

## Data Availability

The data underlying this article are available in the article and in its online supplementary materials.
